# Relationship between Self-Compassion and Uneasiness about the Professional Future among Dental Hygiene Students: A Cross-Sectional Study

**DOI:** 10.1155/2023/8880952

**Published:** 2023-09-27

**Authors:** Maya Izumi, Sumio Akifusa

**Affiliations:** School of Oral Health Sciences, Kyushu Dental University, 2-6-1, Manazuru, Kokurakita-ku, Kitakyushu-shi, Fukuoka 803-8580, Japan

## Abstract

**Purpose:**

To examine the self-compassion of dental hygiene students in Japan and the correlation between their self-compassion and uneasiness about their professional future.

**Methods:**

This study was conducted from May to September 2021. Students were invited from three colleges and one university in Fukuoka Prefecture to take the survey. Participants provided information regarding demographic variables (e.g., gender and year of study) and answered six questions regarding feeling uneasy about one's future, matters of learning, and what might happen after graduation. Self-compassion was assessed using the Japanese short version of the self-compassion scale (SCS), with the positive- and negative-SCS subdomains. Resilience was measured using the bidimensional resilience scale with the subdomains of innate and acquired resilience. For the statistical analysis, participants were divided into the “yes” group, where members felt uneasy and the “no” group, where members did not feel uneasy.

**Results:**

Data were obtained from 464 participants (response rate: 96.3%). The scores of total-, positive-, and negative-SCS were 36 (12–56), 18 (6–29), and 19 (6–30), respectively; 55.2% of students felt uneasy about their professional future. There was a statistically significant difference in scores for positive- and negative-SCS and innate resilience between the “yes” and “no” groups. Binomial logistic regression analysis revealed that when negative-SCS increased by one point, the risk of feeling uneasy about one's professional future was 1.12 times higher (95% confidence interval: 1.07–1.17) after adjusting for resilience and the answers to the remaining five questions.

**Conclusion:**

Our findings suggest that the negative factor of self-compassion is related to increasing the risk of feeling uneasy about one's professional future as a dental hygienist.

## 1. Introduction

Along with advances in dentistry, such as newly developed dental materials and changes in disease structure (e.g., an increase in lifestyles related to disease through the progression of aging), the diversity of knowledge and skills required for dental hygienists is expanding every year. Students in undergraduate dental hygiene programs experience high levels of stress and anxiety in terms of miscellaneous content in the educational curriculum [1]. Students attend classes and practical training simultaneously and are expected to meet the requirements of the curriculum for the dental hygiene profession over the course of 3 years.

Perceived stress negatively impacts nursing students' physical and mental health [2] and hinders academic performance and course completion rates [3, 4]. Moreover, perceived stress is tied to overexhaustion and burnout among dental hygiene students [5–10]. As important psychological characteristics to cope with mental stress, we previously examined the relationship between innate (but not acquired) resilience and self-perceived health in dental hygiene students [11]. Regarding the reasons for dropping out of dental hygienist training schools in Japan, economic reasons comprised 4.9%–6.9% of all reasons, but changing career paths accounted for half [12]. Further, implementing initial-year education and promoting awareness of learning support systems can paradoxically lead to higher dropout rates [13]. Thus, simply enhancing and strengthening educational content and quality alone will not resolve anxiety about the future, and it is necessary to examine the psychological characteristics of dental hygiene students in Japan.

As for new ways of understanding mental well-being, Eastern philosophical thought, especially Buddhism, has often been compared with Western psychology [14]. Unlike Western culture, in Buddhist psychology, it is as essential to feel compassion for oneself as it is for others. Neff defined self-compassion as follows: When we experience pain or anxiety, we have compassion for ourselves (self-kindness); we recognize negative experiences as being shared by humanity (a sense of shared humanity), and we maintain a state of balance by avoiding painful thoughts and feelings (mindfulness) [15]. Many researchers have used the self-compassion scale (SCS), developed by Neff, to explore the relationship between self-compassion and perceived health [16], mental well-being [17], aggression in adolescents [18], and job burnout [19]. We posited that coping with stress would cause dental hygiene students to feel anguish in relation to self-compassion. Thus, we aimed to investigate the relationship between dental hygiene students' anguish (in particular, feeling uneasy about their professional future) and self-compassion.

## 2. Materials and Methods

This study was approved by the Medical Ethics Committee of Kyushu Dental University (No. 21–52). We considered informed consent to have been obtained when the participants agreed to answer the survey following an oral agreement.

The participants were 482 students from four dental hygiene programs in Fukuoka Prefecture. Three schools offer 3-year dental hygiene programs; one university (our affiliated institution) has a 4-year program. Each school's program aligns with the criteria for the national dental hygienist exam. We mailed the survey to each school (except for the university); after checking the returned survey, we remailed responses with incomplete sections to each school. At the university, we collected the survey directly from the participants.

We conducted the survey for this cross-sectional study from May to September 2021. The survey included items regarding demographic variables such as gender, the student's year of study, and six questions about learning issues or what might happen after graduation:

(Q1) I want to work as a dental hygienist in the future.
(Q2) I will make a good dental hygienist.
(Q3) Practical training will be useful in the future.
(Q4) The lecture content will be useful in the future.
(Q5) I feel uneasy about my professional future as a dental hygienist.
(Q6) It is hard for me to learn at school.

The participants could choose from the responses of *not at all*, *not much*, *neither*, *frequent*, or *always*.

To assess psychological resilience, we employed the bidimensional resilience scale (BRS), which was developed to measure innate and acquired resilience [11, 20]. The BRS consists of 21 questions. Questions 1–12 test innate resilience, and questions 13–21 test acquired resilience. Each question is rated on a 5-point Likert scale, where higher scores imply greater resilience. The total possible score ranges from 21 to 105.

To assess self-compassion, we used the short form of the Japanese version of the SCS [21–23]. The SCS consists of 12 questions. Each question is rated on a 5-point Likert scale. The SCS is divided into two subdomains: positive- and negative-SCS. Positive-SCS contains six questions on self-kindness, a shared sense of humanity, and mindfulness. Negative-SCS is composed of six questions regarding self-judgment, isolation, and over-identification. The total possible score for each subdomain ranges from 6 to 30. When calculating the total score of the SCS, the score of positive-SCS was summed up with the remainder of 36 minus negative-SCS (total SCS = positive-SCS + (36 – negative-SCS)). The total possible score ranges from 12 to 60.

The primary outcome was to evaluate the self-compassion of dental hygiene students in Japan. The secondary outcome was to examine the correlation between self-compassion and uneasiness about one's professional future.

We calculated Cronbach's *α* to determine the internal consistency regarding the reliability of each scale. Values are represented as the median (minimum–maximum) for continuous variables and as the number (%) for categorized variables. To analyze the statistical differences of scores for each scale (e.g., SCS and BRS) based on the answers given to each question (e.g., Q1–Q6), we divided the participants into two groups: the “no” group (options for answers: *not at all*, *not much*, *neither*) and the “yes” group (options for answers: *frequent*, *always*). We evaluated the significance of the differences between the groups using the Mann–Whitney *U* test. We appraised the correlation between the two factors using Spearman's rank correlation coefficient. To establish which variables were related to feeling uneasy about one's professional future, we employed Q5. We also performed binomial logistic regression analysis by allowing dichotomized answers for Q1–Q4 and Q6 (positive-SCS, negative-SCS, innate resilience, acquired resilience). To analyze the collinearity of the explanatory variables for binomial logistic regression analysis, we assessed the variance inflation factor. We performed all analyses using SPSS v28 (IBM, Inc.). All statistical comparisons were two-sided, and we considered a <5% error to be statistically significant.

## 3. Results

We recruited 464 students (response rate: 96.3%). Values of Cronbach's *α* for the SCS, positive-SCS, negative-SCS, BRS, innate resilience, and acquired resilience were 0.627, 0.721, 0.853, 0.833, 0.760, and 0.855, respectively.  Table 1 shows the participants' characteristics. All participants were women with a median age of 19 (18–50) years.  Table 2 presents the numbers of each answer for Q1–Q6. Unexpectedly, the proportion of students who answered *neither* for Q2 was 51.1%, and for the “yes” group, it was 28.5%, suggesting that only one out of four students felt they would make a good dental hygienist. Likewise, the responses of the “yes” group (55.2%) to Q5 dominated that of the “no” group (44.8%), indicating that more than half of the students felt uneasy about their professional future.  Table 3 presents the correlation between each subdomain of the SCS and BRS. Positive-SCS was positively correlated with innate and acquired resilience. Negative-SCS was inversely correlated with all other subdomains with statistical significance; however, there was a poor correlation efficiency between negative-SCS and positive-SCS at −0.256. Similarly, the correlation between negative-SCS and acquired resilience was poor because it was −0.168, below 0.3.

Next, to investigate which psychological features were associated with learning issues and self-recognition (assessed by Q1–Q6), we compared the scores of the SCS (Table 4) and BRS (Table 5) between the “yes” and “no” groups for each question. For Q1 (the desire to work as a dental hygienist) and Q3 (the meaning of practical training), only acquired resilience had a statistically significant difference. For Q4 (the meaning of the lecture), negative-SCS and acquired resilience had a statistically significant difference. For Q2 (thinking one will make a good dental hygienist), the scores of all scales of the “yes” group were higher than those of the “no” group, except for negative-SCS. For Q5 (feeling uneasy about one's professional future), the scores of SCS and innate resilience for the “no” group were higher than those of the “yes” group. For Q6 (difficulty learning at school), SCS and its subdomains had a statistically significant difference. Since almost half of the students felt uneasy about their professional future, we performed binomial logistic regression analysis to assess the correlated factors for feeling uneasy about one's professional future. As outlined in Table 6, together with Q2, Q4, and Q6, negative-SCS was correlated with feeling uneasy about one's professional future (B ± SE: 0.11 ± 0.02, odds ratio: 1.12 (95% confidence interval: 1.07–1.17), *p* < 0.001).

## 4. Discussion

The binomial regression analysis indicated that feeling uneasy about one's professional future was statistically significantly correlated with negative-SCS but not positive-SCS or resilience. In the present study, when negative-SCS increased by one point, feeling uneasy about one's professional future was 1.12 times higher. Given that the scores of negative-SCS ranged from 6 to 30 among the participants, negative-SCS could have a significant impact on feeling uneasy about one's professional future. Negative-SCS is reflected in aspects related to self-judgment, isolation, and overidentification. Previous research has demonstrated that self-compassion increases one's perceived competence and intrinsic motivation to learn [24]. Conversely, negative-SCS is correlated with anxiety and depression [14]. To address the issue of anxiety and depression among college students, intervention studies on mindfulness and compassion literacy have been implemented [25–27]. In one study, attending a seminar on compassion improved mindfulness, (self-)compassion, and salivary alpha-amylase as a stress indicator [27]. Another 8-week mindfulness meditation intervention delivered via a consumer-based mobile app called Calm significantly changed perceived stress, mindfulness, and self-compassion [25]. A recent narrative review showed that mindfulness meditation reduced anxiety in 82% of studies and stress in 74% of studies [28]. Interestingly, many studies have examined the effect of yoga on self-compassion [29–32]. A brief 1-hr yoga intervention improved mindfulness in dental and dental hygiene students [32]. An 11-week program of yoga and meditation with a neuroscience didactics intervention enhanced self-regulation and self-compassion among first- and second-year medical students with statistical significance [33]. Thus, the evidence described above suggests that boosting self-compassion literacy and yoga practice may increase mental well-being by enhancing self-compassion.

Neff [21], who developed the original and short form of the SCS, recommended analyzing the total sum, positive-, or negative-SCS, when using the short form. In the present study, Cronbach's *α* for the total score of the SCS was 0.627, indicating possibly insufficient internal consistency in terms of reliability. Thus, we adopted the subdomains of the SCS but not the total SCS to conduct binomial logistic regression. Positive-SCS can be divided into self-kindness, a shared sense of humanity, and mindfulness, while negative-SCS can be divided into self-judgment, isolation, and over-identification. However, it is not recommended to analyze each factor because of the limited number of items that comprise each factor [21].

The proportion of the “no” group regarding one's perceived aptitude as a dental hygienist (assessed by Q2) was 71.6% because the “no” group included participants who selected *neither*, accounting for 51.1%. The “yes” group had greater positive self-compassion, less negative self-compassion, and greater innate and acquired resilience compared to the “no” group (Tables 3 and 4). Academic motivation, assessed using the Academic Motivation Scale, was significantly related to self-compassion and resilience among UK nursing students [34] and Indonesian university students [35], thereby supporting our results. To maintain and improve academic motivation, teaching staff should pay attention to students' self-compassion and resilience.

This study has some limitations. First, because it is a cross-sectional study, the causal link between psychiatric features and the answers for Q1–Q6 is inconclusive. To investigate the influence of self-compassion on emotions or mental well-being, further research on educational interventions, such as attending a mindfulness course, should be conducted. Second, all participants were Japanese women. Importantly, there are cultural differences in the rating scale values of the SCS [36]. The rating scale values of the SCS in Thailand, a Buddhist country, dominate those of the United States, and those of Taiwan are the lowest compared to other countries. A study involving the SCS in Japan revealed that the rating scale values of Japan are similar to those of Taiwan [23]. In Japan, similar to Taiwan, the proportion of the Buddhist population is not dominant. In addition, Japanese people tend to accept self-criticism as a feature leading to self-improvement. Their characteristics are similar to those of the Taiwanese [23]. Third, we did not collect religious information. In Japan, 31% of the participants stated that they believed in Buddhism, while 53% said they followed a certain faith or were religious [37]. Future research on the effect of religion on self-compassion is necessary. Fourth, this study mixed university students following a 4-year curriculum and vocational students in a 3-year curriculum. Most vocational school graduates work for dental clinics. In contrast, while half of university graduates work for dental clinics, the other half work in hospitals, and a few are employed by companies. We cannot deny that these differences in employment choices may impact anxiety about the future. Fifth, we did not collect data related to participants' work experience. This omission constitutes a limitation of our study as these factors could influence the outcomes and interpretations of our findings. In future research, it will be essential to gather such demographic information to provide a more comprehensive understanding of the variables under investigation.

## 5. Conclusion

We found the negative factors of self-compassion to be correlated with feeling uneasy about one's professional future among Japanese dental hygiene students. To improve students' academic motivation and mental well-being, it is necessary to assess their self-compassion.

## Figures and Tables

**Table 1:** Sociodemographic characteristics of the participants (*N* = 464).

Variable	Number (%)
Sex	
Female	464 (100)
Student's year of study	
1st	170 (36.6)
2nd	138 (29.7)
3rd	133 (28.7)
4th (university)	23 (5.0)
	Median (minimum–maximum)
Age	19 (18–50)
SCS	36 (12–56)
Positive-SCS	18 (6–29)
Negative-SCS	19 (6–30)
BRS	74 (38–140)
Innate resilience	42 (16–96)
Acquired resilience	32 (13–45)

*Note*: SCS, self-compassion scale; BRS, bidimensional resilience scale.

**Table 2:** Numbers of each answer for each question.

Questions		Answer				
		No			Yes	
		Not at all	Not much	Neither	Frequent	Always
Q1	I want to work as a dental hygienist in the future	68 (14.6)			396 (85.4)	
		2 (0.4)	12 (2.6)	54 (11.6)	193 (41.6)	203 (43.8)

Q2	I will make a good dental hygienist	332 (71.6)			132 (28.4)	
		10 (2.2)	85 (18.3)	237 (51.1)	113 (24.4)	19 (4.1)

Q3	Practical training will be useful in the future	25 (5.4)			439 (94.6)	
		1 (0.2)	4 (0.9)	20 (4.3)	143 (30.8)	296 (63.8)

Q4	The lecture content will be useful in the future	79 (17.0)			385 (83.0)	
		2 (0.4)	13 (2.8)	64 (13.8)	218 (47.0)	167 (36.0)

Q5	I feel uneasy about my professional future as a dental hygienist	208 (44.8)			256 (55.2)	
		23 (5.0)	74 (15.9)	111 (23.9)	179 (38.6)	77 (16.6)

Q6	It's hard for me to learn at school	397 (85.6)			67 (14.4)	
		80 (17.2)	156 (33.6)	161 (34.7)	51 (11.0)	16 (3.4)

**Table 3:** Correlation between each subdomain of the SCS and BRS.

		#1	#2	#3	#4
#1	Positive-SCS	1			
#2	Negative-SCS	−0.256^*∗∗*^	1		
#3	Innate RS	0.360^*∗∗*^	−0.329^*∗∗*^	1	
#4	Acquired RS	0.452^*∗∗*^	−0.168^*∗∗*^	0.425^*∗∗*^	1

*Note*:  ^*∗∗*^*p*-Value < 0.01.

**Table 4:** Difference in scores on the SCS and its subdomains based on the participants' response to each question.

	*N*	SCS	Positive-SCS	Negative-SCS
Q1				
No	68	35.5 (18–51)	18 (9–25)	18.5 (8–30)
Yes	396	36 (12–56)	18 (6–29)	19 (6–30)
*p*-Value		0.619	0.258	0.905
Q2				
No	332	35 (12–56)	18 (6–27)	20 (6–30)
Yes	132	39 (16–56)	20 (8–29)	17 (6–30)
*p*-Value		<0.001	<0.001	<0.001
Q3				
No	26	35 (18–49)	17.5 (9–24)	19 (11–28)
Yes	428	36 (12–56)	18 (6–29)	19 (6–30)
*p*-Value		0.465	0.311	0.943
Q4				
No	79	35 (15–51)	18 (9–25)	19 (6–30)
Yes	385	36 (12–56)	19 (6–29)	19 (6–30)
*p*-Value		0.116	0.022	0.561
Q5				
No	208	38 (24–56)	19 (6–27)	17 (6–30)
Yes	256	34 (12–56)	18 (6–29)	20 (6–30)
*p*-Value		<0.001	0.017	<0.001
Q6				
No	397	36 (15–56)	18.5 (6–29)	18.5 (6–30)
Yes	67	34 (12–56)	18 (6–26)	20 (6–30)
*p*-Value		<0.001	0.017	<0.001

*Note*: SCS, self-compassion scale.

**Table 5:** Difference in scores on the BRS and its subdomains based on the participants' response to each question.

	*N*	BRS	Innate RS	Acquired RS
Q1				
No	68	73.5 (51–135)	42 (24–96)	29 (20–43)
Yes	396	75 (38–140)	42 (16–95)	32 (13–45)
*p*-Value		0.695	0.810	0.001
Q2				
No	332	72 (38–137)	41 (16–96)	31 (13–44)
Yes	132	82 (54–140)	48 (27–95)	34 (21–45)
*p*-Value		<0.001	<0.001	<0.001
Q3				
No	26	73.5 (51–111)	42 (30–77)	30 (21–38)
Yes	428	74.5 (38–140)	42 (16–96)	32 (13–45)
*p*-Value		0.485	0.781	0.034
Q4				
No	79	73 (43–140)	42 (20–95)	30 (21–45)
Yes	385	75 (38–137)	42 (16–96)	32 (13–44)
*p*-Value		0.045	0.271	0.002
Q5				
No	208	75 (38–140)	43 (24–95)	32 (14–45)
Yes	256	74 (43–135)	41.5 (16–96)	32 (13–43)
*p*-Value		0.098	0.033	0.294
Q6				
No	397	75 (38–140)	42 (20–96)	32 (13–45)
Yes	67	71 (48–127)	42 (16–90)	32 (21–44)
*p*-Value		0.094	0.153	0.264

**Table 6:** Binomial logistic regression analysis of the responses to Q5 (feeling uneasy about one's future as a dental hygienist).

Variables	*B* ± SE	OR (95% CI)	*p*-Value	VIF
Age	0.00 ± 0.03	1.00 (0.95–1.06)	0.192	1.021
Q1 (reference: no)	−0.07 ± 0.31	1.07 (0.58–1.99)	0.766	1.191
Q2 (reference: no)	−0.77 ± 0.25	2.17 (1.33–3.52)	0.004	1.226
Q3 (reference: no)	0.40 ± 0.45	0.67 (0.28–1.61)	0.378	1.077
Q4 (reference: no)	0.84 ± 0.29	0.43 (0.24–0.76)	0.005	1.094
Q6 (reference: no)	1.32 ± 0.35	0.27 (0.14–0.52)	<0.001	1.057
Positive-SCS	−0.03 ± 0.03	0.98 (0.91–1.04)	0.492	1.357
Negative-SCS	−0.11 ± 0.02	0.89 (0.86–0.94)	<0.001	1.137
Innate RS	0.00 ± 0.01	1.00 (0.99–1.02)	0.815	1.289
Acquired RS	0.01 ± 0.03	1.01 (0.97–1.07)	0.582	1.406

*Note*: B, regression coefficient; SE, standard error; OR, odds ratio; 95% CI, 95% confidential interval; VIF, variance inflation factor; SCS, self-compassion scale; RS, resilience scale.

## Data Availability

The data used to support the findings of this study are available from the corresponding author upon request.
